# Defining Nontuberculous Mycobacterium Surgical Site Infections at a Tennessee Ambulatory Surgery Center with NHSN Surveillance Protocol

**DOI:** 10.1017/ash.2025.407

**Published:** 2025-09-24

**Authors:** Jordan Morris, Ashley Gambrell, Simone Godwin

**Affiliations:** 1 Tennessee Department of Health; 2 Tennessee Department of Health; 3 Tennessee Department of Health

## Abstract

**Background:** Procedures performed at Ambulatory Surgical Centers (ASCs) have increased over the last decade in the United States. In Tennessee, surgical site infection (SSI) outbreaks in ASCs have been increasingly detected. Still, there is no mandated SSI reporting for ASCs through the National Healthcare Safety Network (NHSN) as there is for Acute Care Hospitals (ACHs). In 2023, the Tennessee Department of Health’s Healthcare-Associated Infections (TDH HAI/AR) program responded to an outbreak of 14 nontuberculous mycobacteria (NTM) periprosthetic joint infections at an ASC. Despite extrapulmonary NTM being a reportable condition in Tennessee, detection of this outbreak was delayed due to gaps in reportable conditions practices at this ASC. Here, we evaluate how NHSN reporting could have impacted the surveillance and detection of infections for this investigation. **Methods:** Extrapulmonary NTM cases were detected through clinical laboratory and provider reporting. Chart abstractions were performed for cases by HAI/AR epidemiologists using a tool adapted from the Centers for Disease Control and Prevention (CDC). Infections were evaluated using standardized 2023 and 2024 National Healthcare Safety Network (NHSN) definitions depending on the infection date of event. **Results:** Initial reporting of cases was as mentioned above, resulting in five cases reported together in June 2023, two months after the first positive specimen. Eight (57%) cases met the NHSN definition for Surgical Site Infections (SSIs); four (29%) cases met the criteria for Deep Incisional SSIs, and four (29%) met the Organ/Space SSI. Six cases (43%) were not detected within the 90-day surveillance window; however, three of these cases had documented evidence of superficial infection within those 90 days. **Conclusions:** Despite its slow infection progression, most NTM infections in this outbreak would have been detected through NHSN surveillance. Even in cases where NHSN SSI criteria were not met, reviewing records and entering data within the NHSN framework may have facilitated faster facility-level detection. Although the nature of NHSN reporting is not suited for rapid detection of outbreaks, the standardized definitions, regular records reviews, and established data entry system would benefit ASC surveillance such as the facility described here, which had no formal mechanism for tracking infections. Additionally, the collection of summary data required through NHSN would better identify reporting gaps prior to outbreak occurrences. The availability of SSI data for ASCs would help public health authorities identify and assist facilities in assessment and prevention activities. Patient safety would thus likely benefit from enhancing surveillance of ASCs through voluntary or

